# PlomBOX: a low cost bioassay for the sensitive detection of lead in drinking water

**DOI:** 10.1038/s44172-024-00337-7

**Published:** 2025-01-07

**Authors:** A. Dias, M. Alvarez, Y. Gándola, A. Deisting, E. Alba Posse, H. Arnaldi, H. Asorey, X. Bertou, A. Colque, F. Favela-Pérez, J. Gasulla, M. Gómez Berisso, J. O. Guerra-Pulido, J. Lipovetzky, J. Lobera, M. B. Lovino, L. Marpegan, D. Martín, S. Mejía Muñoz, J. Monroe, A. D. Nadra, R. Pregliasco, G. Rumi, A. Rossen, M. Tallis, A. Thompson, M. Triana, M. L. Vazquez Miranda

**Affiliations:** 1 Department of Physics, Royal Holloway, University of London, Egham, UK; 2 Departamento de Fisiología, Biología Molecular y Celular, Facultad de Ciencias Exactas y Naturales, Instituto de Biociencias, Biotecnología y Biología Traslacional (iB3), Consejo Nacional de Investigaciones Científicas y Técnicas (CONICET), Universidad de Buenos Aires (UBA), Buenos Aires, Argentina; 3 Institut für Physik & Exzellenzcluster PRISMA+, Johannes Gutenberg-Universität Mainz, Mainz, Germany; 4 Centro Atómico Bariloche and Instituto Balseiro, Comisión Nacional de Energía Atómica (CNEA), Consejo Nacional de Investigaciones Científicas y Técnicas (CONICET), Universidad Nacional de Cuyo (UNCUYO), San Carlos de Bariloche, Argentina; 5 Instituto de Tecnologías en Detección y Astropartículas, Comisión Nacional de Energía Atómica (CNEA), Consejo Nacional de Investigaciones Científicas y Técnicas (CONICET), Universidad Nacional de San Martín (UNSAM), Buenos Aires, Argentina; 6 Facultad de Arquitectura, Diseño y Urbanismo, Universidad de Buenos Aires, Buenos Aires, Argentina; 7 Instituto de Ciencias Nucleares, Universidad Nacional Autónoma de México, CDMX, Mexico; 8 Department of Physics, University of Oxford, Oxford, UK; 9 Subgerencia Laboratorio de Calidad de Agua, Instituto Nacional del Agua, Buenos Aires, Argentina; 10 Facultad Regional Avellaneda, Universidad Tecnológica Nacional, Buenos Aires, Argentina

**Keywords:** Assay systems, Environmental impact

## Abstract

This paper reports the design of a biosensor for sensitive, low-cost measurement of lead in drinking water. The biosensor uses a genetically-modified strain of *Escherichia coli*, which serves as both signal amplifier and reporter of lead in water, measured via colour change. We developed the PlomBOX measurement platform to image this colour change and we demonstrate its capability to detect concentrations as low as the World Health Organisation upper limit for drinking water of 10 ppb. Our approach does not require expensive infrastructure or expert operators, and its automated sensing, detection and result visualisation platform is user-friendly and robust compared to existing lead biosensors—critical features to enable measurement by non-experts at the point of use.

## Abstract (web summary)

Adriana Dias and co-authors report a low-cost bioassay for the detection of low concentrations of lead in drinking water. The approach uses a genetically modified strain of *Escherichia coli*, which serves as both signal amplifier and reporter of lead in water, measured via colour change.

## Introduction

Lead is one of ten chemicals that the World Health Organisation (WHO) has identified as a major public health concern^1^. Lead has been widely used in manufacturing and anthropogenic impacts on the environment have resulted in the presence of lead in drinking water, food and air. Lead exposure causes a myriad of diseases accounting for an estimated 1 million deaths per year globally^2^. Lead toxicity affects both adults and children, although children are particularly vulnerable as their soft tissues absorb excess lead^3^. Lead produces nefarious effects on the human body: it interferes with enzymes that help produce vitamin D and maintain the integrity of cell membranes and it damages cells structures, including DNA^4,5^. Chronic lead poisoning is associated with a reduced life expectancy and the impact of lead exposure is estimated to be between 2,000,000 and 4,800,000 DALYs (Disability-adjusted life years) annually^6^. One DALY can be thought of as one year of healthy life lost, or alternatively a measure of the difference between health in the current situation and one where the population was able to age free of disease and disability. Even a lead intake of concentrations as low as 1 *μ*g g^−1^ (1000 ppb) over a prolonged period is hazardous to humans^7^.

A major source of lead intake comes from drinking water delivered through lead pipes, or pipes soldered with lead. The WHO upper limit on lead in drinking water is 10 ppb, although no level has been shown to be safe for humans. Lead in water measurements employ expensive and specialised detection techniques, such as atomic absorption spectrometry. Besides the high investment cost of the equipment, such assays require specialised technical personnel for operation and analysis. Therefore, assays are not widely available and the cost per assay is prohibitive in many parts of the world. Low and middle income countries are mostly at risk from lead exposure, with an estimated 26 million people being affected globally^8^. Given the high public health burden, simple and affordable lead detection techniques are needed.

There are microorganisms that adapted to toxic heavy metals in the environment by developing transcriptional regulators sensitive to metal ions such as Hg^2+^, Cd^2+^, Pb^2+^. The MerR family is one of the main families of metal-response proteins characterised by high sensitivity and selectivity for the target metal ion^9^. Many whole-cell biosensors have been developed based on the metalloregulatory proteins of the MerR family using fluorescent, luminescent and enzymatic reporters. But many of these developed biosensors are not able to detect lead at the maximum allowed by the WHO^10–16^. The use of an enzymatic method as a reporter compared to other methods has the advantage of generating enhanced sensitivity of the system due that a low enzyme production is able to amplify detectable signal^15^.

In this study, we developed a genetically modified bacterium containing an enzymatic reporter (LacZ-*α*), a MerR family transcriptional regulator (PbrR) and a transport protein that facilitates Pb^2+^ entry into the cell, PbrT, which has not been included in previously developed biosensor genetic constructions. The idea behind including a lead transporter is that it could increase the uptake of Pb^2+^ and thus increase the sensitivity of the system^15^. Notably, when dealing with very low concentrations, it is not only important the modules used, but also its specific implementation, as in our experience small variants in biosensor design were differently sensitive to Pb^2+^ levels.

Various lead sensors have been developed based on small molecular probes, peptides, proteins and nucleic acids^17^. The biosensors published so far, while promising, often suffer from complicated fabrication of sensing materials, expensive instruments for detection or complicated techniques for result visualisation^18–20^. These biosensors require trained and knowledgeable people to use them, which means that these biosensors are not user-friendly by non-experts. To make a sensor user-friendly and capable of measuring lead contamination at the point of use, it should be portable, without requiring bulky analytical instruments or precise handling of small volumes of liquid. At time of writing, a biosensor with all the desired characteristics has not yet been demonstrated.

This paper describes the design of a lead biosensor bacteria and development of a low-cost, portable measurement platform, called PlomBOX. PlomBOX facilitates lead detection in drinking water at the point of use, by non-experts, helping reduce lead intake through contaminated water. PlomBOX consists of: (i) a genetically modified strain of *Escherichia coli* responsive to lead; (ii) an imaging system to measure the bacteria response, based on commercial off-the-shelf components; and, (iii) a mobile phone application—the PlomApp—that acquires data from the PlomBOX and returns the result of the lead concentration in a water sample to the user. This measurement platform costs approx. 20 USD and is reusable, except for the lead-sensing bacteria (introduced as a disposable cartridge). The PlomBOX could easily be adapted to detect other water pollutants by changing the bacterial biosensor. Additionally, other developed whole-cell biosensors expressing other reporters, different to the one used by PlomBOX, can be used by adjusting the measurements for the different colours, as long as the intensity of the emitted signal is similar to ours. All this makes the PlomBOX measurement platform versatile.

We report the results of lead assay in water using the PlomBOX, showing high selectivity and sensitivity to the presence of lead at the WHO 10 ppb limit in both controlled laboratory reference samples and in a household drinking water monitoring campaign in Buenos Aires, carried out in collaboration with the National Water Institute.

## Results

### PlomBOX design

**Fig. 1:**
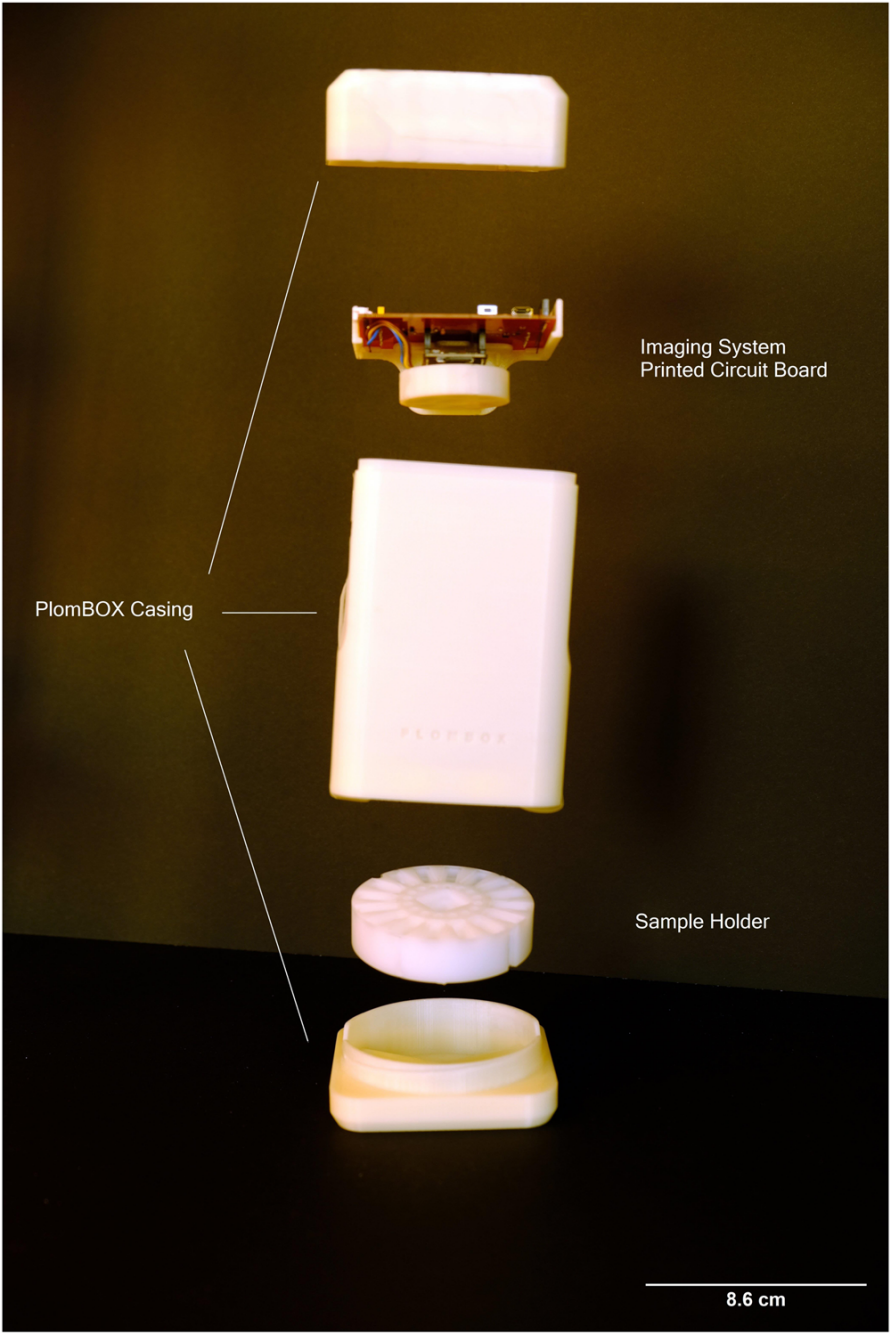
PlomBOX casing and internal components. Photo of the PlomBOX’s casing design, with the imaging system visible at the top.

The components of the PlomBOX comprise the biosensor bacteria, the imaging system inside a mechanical housing, seen in Fig. 1, and the data acquisition application.

#### Biosensor

**Fig. 2:**
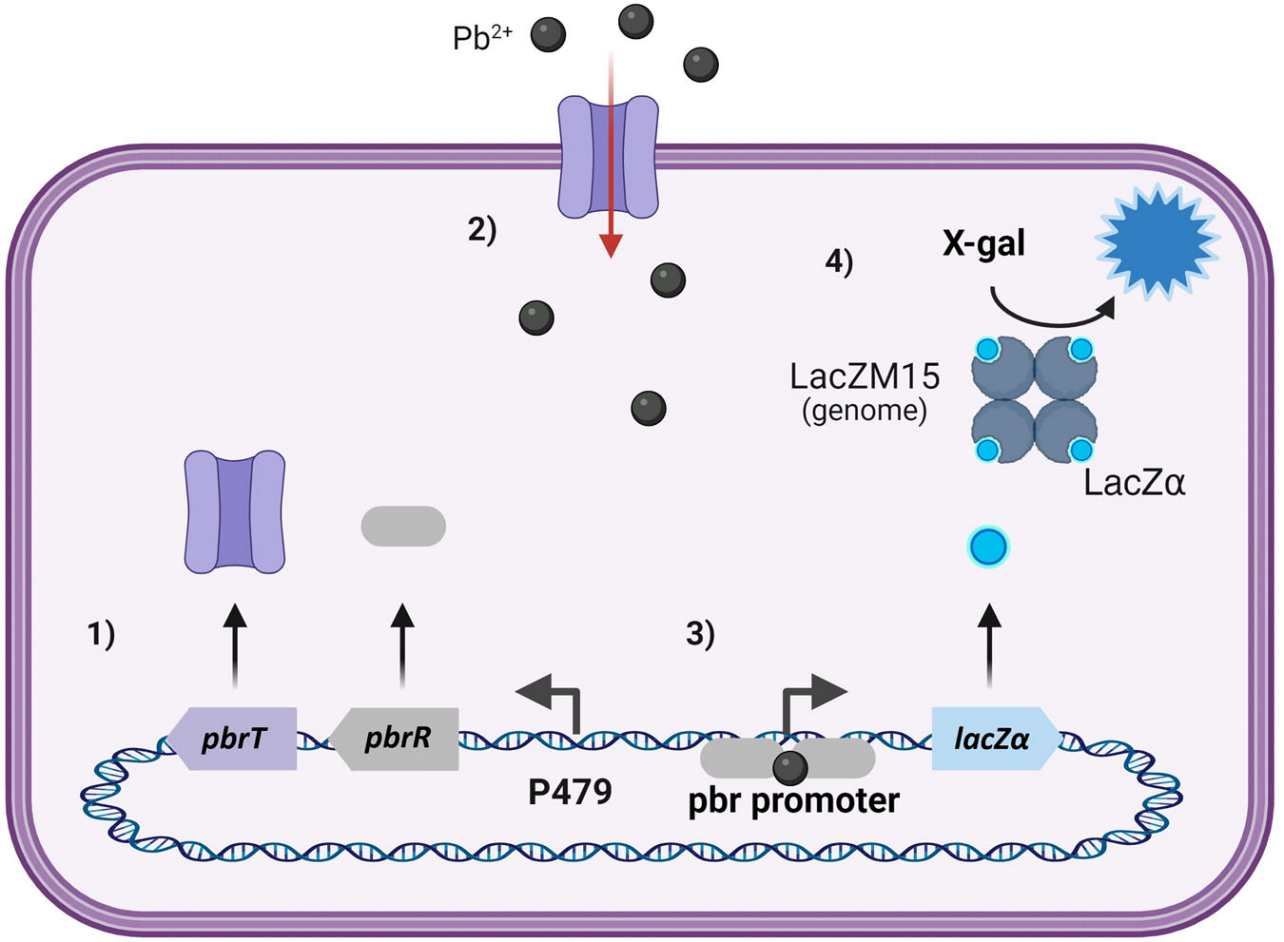
Schematic representation of the biosensing process. PbrR and PbrT proteins are constitutively expressed (1). When lead is present in the extracellular media, PbrT facilitates its transport to the bacteria cytoplasm (2). Pb^2+^ binds to PbrR producing the expression of the LacZ*α* gene (3). The active *β*-galactosidase tetramer is formed, X-gal is cleaved and an intense blue colour is produced, indicating the presence of lead.

The PlomBOX bacteria is an *E. coli* DH5*α* strain bearing the lead-sensing genetic construction based on the *pbr* operon, placed on a plasmid of high copy number^21^. This construction codifies a homodimeric protein of *Klebsiella pneumoniae* (PbrR), which acts as a transcriptional regulator and has been shown to specifically bind to Pb^2+^, and a regulatory zone upstream of the LacZ*α* fragment gene, as the reporter—Fig. 2^10,13^. In addition, we included a lead transporter protein (PbrT) of *Cupriavidus metallidurans* to facilitate the intracellular Pb^2+^ increase in concentration. Both PbrR and PbrT proteins are placed under the intermediate strength constitutive promoter P479, whereas the LacZ*α* fragment expression is regulated by a lead sensitive promoter/operator^22^. When lead is present in the extracellular media, it is internalised into the cytoplasm, where it binds to PbrR, inducing a conformational change in the homodimer and modifying its affinity for the promoter/operator^23^. As a consequence, the LacZ*α* fragment is expressed. The *β*-galactosidase enzyme is the reporter protein. This enzyme is functional when it is composed of four identical *β*-galactosidase polypeptides. DH5*α* strain bears the *lacZΔM15* mutation that impairs the *β*-galactosidase tetramer formation^24^. In this way, only in the presence of lead is the LacZ*α* fragment expressed and the complete monomers can tetramerise to form a functional *β*-galactosidase enzyme. This tetramer can then cleave the glycosidic bond in the substrate X-gal to produce galactose and an intense blue signal that is easy to identify and quantify.

#### Imaging System

The imaging system consists of a low-cost CMOS, hosted on an electronics development board with a microprocessor that controls the illumination of the water samples by 3 colour LEDs, and communicates with the PlomApp via Bluetooth^25^. The PlomBOX mechanical housing encloses the imaging system and the water sample holder, and is designed to be 3D printed. In^26^ we have collected the material necessary to print a PlomBOX.

The PlomBOX is prepared as described in the Measurement duration section. Once the sample holder is loaded and the PlomBOX is assembled and powered, a time series of images of the sample holder are acquired. The sample holder can accommodate 16 samples (*cf*. Figures 1 and 8). Of these 16 wells four to six are used for the calibration curve samples with known lead concentrations. Image data are acquired from the PlomBOX using a mobile phone application, PlomApp, developed in Android Studio using Java. The application interacts with the firmware on the development board to control the PlomBOX, acquiring image data via Bluetooth and collecting meta-data from the smartphone’s geo-location sensors. Once received, the image and meta-data are sent to a remote server via Message Queuing Telemetry Transport (MQTT) protocol. The server analyses the data and returns the lead result per sample to the PlomApp via MQTT.

### Data analysis

To evaluate the PlomBOX sensing performance, we tested the response of the genetically modified bacteria to different lead concentrations, under controlled laboratory conditions. Measurements were taken using multiwell plates and milli-Q ultrapure water (grade III, ISO 3696) with lead concentrations of 0, 10, 30 and 100 ppb, prepared from a lead standard by serial dilutions. Due to variability in bacterial growth caused by different growth conditions expected in the field, such as temperature, batch, etc, the PlomBOX records a lead calibration curve (termed lead curve in the following) with each assay to account for these differences. Results are presented in the Lead curves measured by qualified Reference Laboratories section.

We assessed the PlomBOX performance under real-world conditions by measuring drinking water samples from selected houses with lead pipes in Buenos Aires. Calibration lead curve samples with lead concentrations of 0, 10, 15, 20, 50 and 100 ppb were prepared using de-ionised water with weighted lead powder. We use de-ionised water for these lead curves as this is more accessible. Results are presented in the Comparison with Water Sampling Campaign section.

Both the laboratory and real-world studies showed excellent sensitivity to 10 ppb lead concentration in water samples.

#### Analysis pipeline

The key observable for estimating lead concentration is the colour change vs. time measured in the images acquired by the PlomBOX. Data analysis involves calibrating colour measurements, bacteria response, and measuring the colour change of the water sample to estimate its lead concentration.

##### Colour measurement and calibration

The colours in the sample holder wells are measured from images taken by the PlomBOX stored in Joint Photographic Experts Group (JPEG) format. This format was chosen for its small file size, suitable for Bluetooth transmission to the PlomApp. The JPEG images contain red (R), green (G) and blue (B) colour values for each pixel. The following operations are performed on this image data.

*Colour calibration:* Colour perception depends on illumination conditions, therefore the PlomBOX controls the sample holder’s lighting with red, green, and blue LEDs. Data acquisition begins with an LED calibration set of images to infer calibration constants for each LED, such that the resulting combined image has a proper white balance. This process determines the exposure length for each LED in subsequent images. Details of the calibration are given in the Image Colour and Uniformity Calibration section.

*Flat fielding:* Flat fielding corrects for variation in illumination intensity across the image. This produces a pixel-by-pixel corrected image, as described in the Image Colour and Uniformity Calibration section, removing the effects of heterogeneous illumination^27^.

*Region of interest selection:* After flat fielding, the analysis software uses the opencv-python python library to read a QR code and locate the regions of interest (ROIs) for the bacteria samples, corresponding to each well’s position in the sample holder. The QR code can contain information about the assay date, PlomBOX identifier, sample origin, test configuration, etc. The RGB values for each pixel in each ROI are then averaged, giving each sample well one R, G and B value.

*Colour decomposition:* The colour change analysis uses Hue, Saturation and Value (HSV), calculated from the average RGB values for each ROI, using the pycopy-colorsys python library. HSV is chosen as it closely resembles human colour perception and is quantifiable^28^. Studies showed that saturation (S) provided the maximum measurable change over time for calibration lead samples. HSV value uncertainties are propagated from the RGB uncertainties, obtained by calculating the standard error of the mean for R, G and B values, ${\sigma }_{\overline{j}}$ (*j* ∈ R,G,B), for all pixels in each ROI.

*Calibrations applied to time series data:* After image processing, the analysis pipeline applies several calibrations to produce the saturation value for each ROI with an unknown lead concentration, at each time *t* in the series of images. These calibrations include absolute colour calibration in the HSV colour basis and subtraction of the initial image saturation value; these are described in the Image Data Analysis and Uncertainty Propagation section, together with the related propagation of uncertainties for each calibration step.

##### Sample lead concentration measurement

**Fig. 3:**
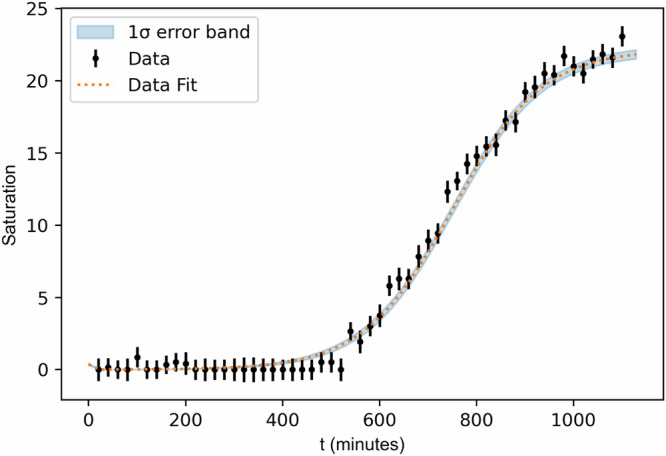
An example of a timelapse fit for one sample, from an assay of water collected in the household water sampling campaign in Buenos Aires. The timelapse data (black data points showing saturation vs time and the uncertainty on the saturation measurement) is shown, together with a fit (orange dashed line), with a 1 *σ* error band in light blue. The ${\chi }_{{{\rm{red}}}}^{2}$ statistic for this fit is 1.02 and the fit returned a final saturation value of 21.8 ± 0.4.

The final step in the analysis entails obtaining a value for the lead in the water samples of unknown concentration. The timelapse of measured saturation vs. *t* is fit to an analytic function modelling gene expression and continuous bacterial growth, described in the Time series data fit function and uncertainty propagation section; an example is shown in Fig. 3. Figure 3 shows the 1 *σ* error band from propagating uncertainties at each step of the measurement and calibration process. The goodness of the fit is quantified with a reduced chi-square statistic (${\chi }_{{{\rm{red}}}}^{2}$). The final saturation value is the best-fit function value (and 1 *σ* uncertainty) evaluated at the end time of the assay.

**Fig. 4:**
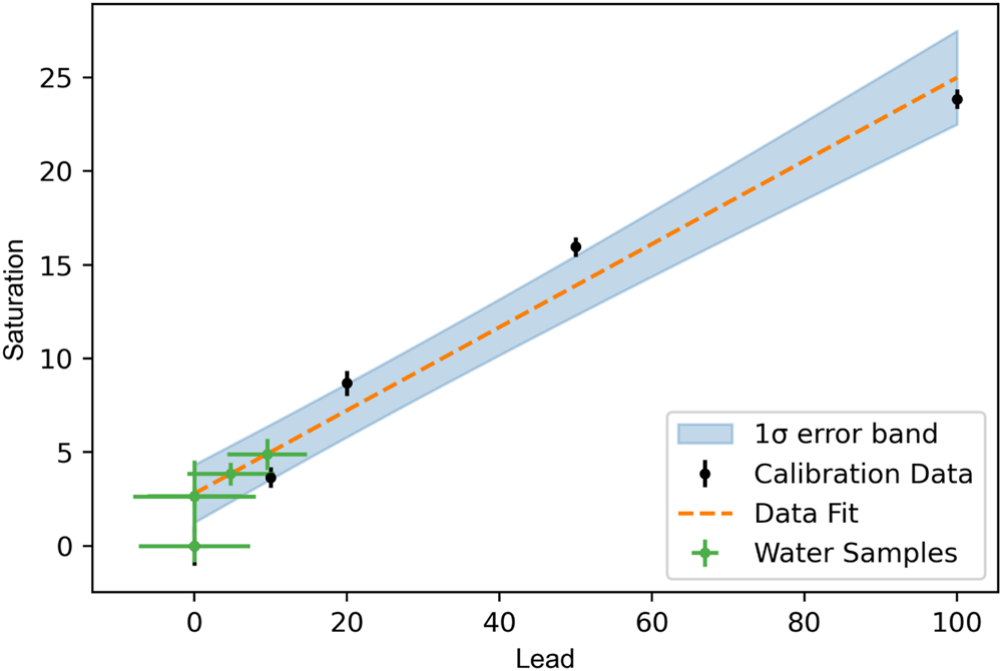
An example for a lead curve fit for one assay, using water collected in the household water sampling campaign in Buenos Aires. A linear fit (orange dashed line) is produced from the lead curve data (with concentrations of 0, 10, 20, 50 and 100 ppb), seen in black. The samples' saturation values are interpolated on this fit to find their lead values in green, with concentrations of 9.5, 4.75 and 0 ppb. Three of the samples returned a concentration of 0 ppb. The fit is shown in orange and the 1 *σ* error band is shown in light blue.

Following the calibration steps described above, a look-up table of the saturation values of the lead curve samples vs. lead concentration is constructed. The same time series fit methodology is applied to the lead curve samples co-measured with the unknown samples. The final saturation values and uncertainties, evaluated from the best-fit function at the end time *t* of the time series, are used to construct the lead curve of saturation vs. lead concentration. This lead curve is fit with a first-order polynomial and the saturation values of the unknown tap water samples are linearly interpolated, returning a value of lead for each of them. The uncertainty contribution from the lead curve fit is propagated into the uncertainty on the final lead value for the unknown sample as described in the Lead curve fits section. Figure 4 shows a lead curve fit, the lead curve data points and the interpolated sample data.

#### Measured performance

The PlomBOX data sets used to assess performance have data quality cuts applied to select assays in which the bacteria successfully grew; for details see Data quality cuts section. Successful growth is determined by saturation reaching 50% of its final value by 1000 min of the assay. Performance is quantified in terms of accuracy at 95% confidence level (CL) as defined in the PlomBOX Accuracy section. PlomBOX performance is benchmarked against measurements by standard reference laboratories and AAS measurements by the Argentina National Water Institute.

##### Lead curves measured by qualified Reference Laboratories

Lead curve samples with 0, 10, 30 and 100 ppb of lead in milli-Q water were prepared by us and measured by qualified Reference Laboratories (National Water Institute, Water Quality Laboratory Sub-management, Experimental Laboratory for Sustainable Technologies). The same samples were then measured in PlomBOX multiple times for each sample. The saturation values for all the elements were interpolated on their respective lead curves and lead values were obtained. The mean of the measured lead values, $\overline{x}$, was calculated, and confidence intervals (CI) were acquired for each lead concentration as ${{\rm{CI}}}=\overline{x}\pm ({t}_{{{\rm{score}}}}\cdot {\sigma }_{\overline{x}}).$ The value of the ${t}_{{{\rm{score}}}}$ for a two-tailed test to assess the 95% CI was chosen according to the number of lead samples included in $\overline{x}$. A ${t}_{{{\rm{score}}}}$ is used here, rather than a ${z}_{{{\rm{score}}}}$, because the sample sizes are lower than the minimum 30 required for a *z*-test.

**Fig. 5:**
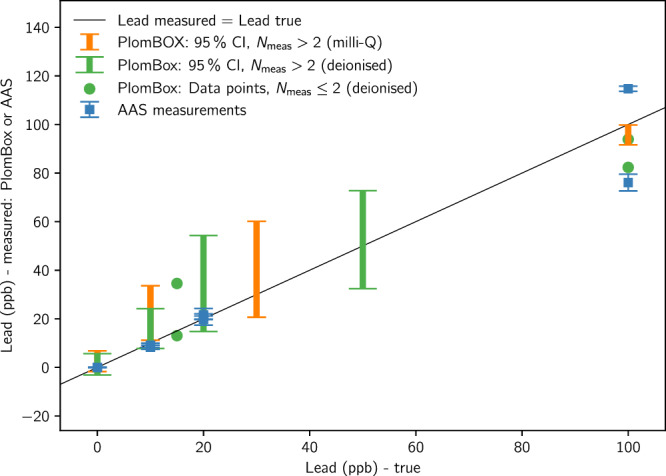
True vs. measured sample lead value, using milli-Q and de-ionised water calibration samples. 95% confidence intervals are shown in coloured bands for all instances where more than 2 samples (*N*_mea_ > 2) were measured. For comparison, reference samples measured by atomic absorption spectroscopy (AAS) are shown. The error-bars on the AAS data points are the standard deviation of the three measurements done for each sample multiplied by the corresponding ${t}_{{{\rm{score}}}}$ (4.303) for 95% CI, to enable a better comparison between PlomBOX and AAS results. The Lead measured = Lead true line has been drawn to guide the reader.

The mean value results showed excellent agreement with the expected values for the lead curve within the 5% uncertainty on the reference laboratory measurement. Figure 5 shows the confidence interval results and Table 1 tabulates these results: there is 95% confidence that the PlomBOX can distinguish between two samples that have concentrations of 0 and  > 10 ppb.

**Table 1:** Table showing accuracy of the PlomBOX by calculating the confidence intervals (CI) for different concentrations of lead, using milli-Q water (Fig. 5)

Lead (ppb)	$\overline{x}$	${\sigma }_{\overline{x}}$	CI (95%)	*p*-value
0	2.53	1.74	4.84	0.20
10	22.39	4.58	12.73	0.04
30	40.39	8.07	22.42	0.25
100	95.70	1.67	4.63	0.04

Here, $\overline{x}$ is the mean of the measured lead values and ${\sigma }_{\overline{x}}$ is the standard error of this mean.

##### Comparison with water sampling campaign

A household water sampling campaign was conducted in Buenos Aires, together with the National Water Institute of Argentina (Instituto Nacional del Agua, INA), as described in the INA Sampling Campaign and Chemical Analysis section. These samples were measured by INA, using atomic absorption spectroscopy (AAS), and by the PlomBOX.

The volume collected in the domestic sampling campaign was insufficient to repeat the assay enough times to assess confidence intervals via multiple measurements in the PlomBOX and thus instead the correlation between the PlomBOX measurement of domestic samples and AAS results is quantified using the Pearson correlation coefficient. The correlation between PlomBOX and AAS measurements, for data passing the data quality cuts (seen in Table 2), is 83%. The correlation coefficient between PlomBOX data passing data quality cuts and the qualified reference laboratory measurements of milli-Q calibration samples is 80%. We designed this biosensor to be sensitive to lead in the range near the WHO limit of 10 ppb; this study shows the range of validity of the PlomBOX measurement is approximately  < 100 ppb.

**Table 2:** Data used to determine PlomBOX’s correlation with AAS measurements (Fig. 5)

Date	Sample number	AAS lead	PlomBOX lead (uncertainty)
01/12/2022	82	5	9.53 (5.23)
01/12/2022	83	9	0 (6.16)
01/12/2022	84	6	0 (8.01)
01/12/2022	87	57	4.75 (5.72)
01/12/2022	90	12	0 (7.28)
14/12/2022	101	7	1.08 (5.14)
14/12/2022	102	7	10.29 (4.91)
14/12/2022	103	98	42.31 (9.7)

The dates of the assays are shown, together with the identification number of the sample (Sample Number) and the lead values, in ppb, obtained by AAS and PlomBOX measurements. Note that the uncertainties on AAS are in the range of 0.1 ppb to a few ppb.

We further quantify the de-ionised water calibration samples similarly to milli-Q as described above. The 95% confidence interval for multiple measurements of the de-ionised water lead curve samples is shown in Fig. 5, and gives consistent results with the milli-Q test. The 0 and 10 ppb 95% CI are statistically distinguishable and thus there is 95% confidence that the PlomBOX can distinguish between two samples that have concentrations of 0 and  > 10 ppb, using de-ionised water. Table 3 tabulates these results. Note that only two assays with 15 ppb and 100 ppb of lead passed the cuts, which is not enough to calculate meaningful CI for this concentration. Therefore, for these two concentrations data points are shown in Fig. 5 as opposed to CIs.

**Table 3:** Table showing accuracy of the PlomBOX by calculating the confidence intervals (CI) for different concentrations of lead, using de-ionised water

Lead (ppb)	$\overline{x}$	${\sigma }_{\overline{x}}$	CI (95%)	*p*-value
0	1.25	1.25	5.37	0.42
10	15.97	2.33	10.03	0.12
20	34.53	5.63	24.22	0.12
50	52.58	5.75	24.72	0.70

Here, $\overline{x}$ is the mean of the measured lead values and ${\sigma }_{\overline{x}}$ is the standard error of this mean. We did not calculate a CI for the two samples of 15 ppb and 100 ppb of lead, as CIs for two data points are not meaningful. The PlomBOX measurements for the 15 ppb samples were: 34.5 ppb and 13 ppb. For the 100 ppb samples the values were: 93.9 ppb and 82.3 ppb.

**Fig. 6:**
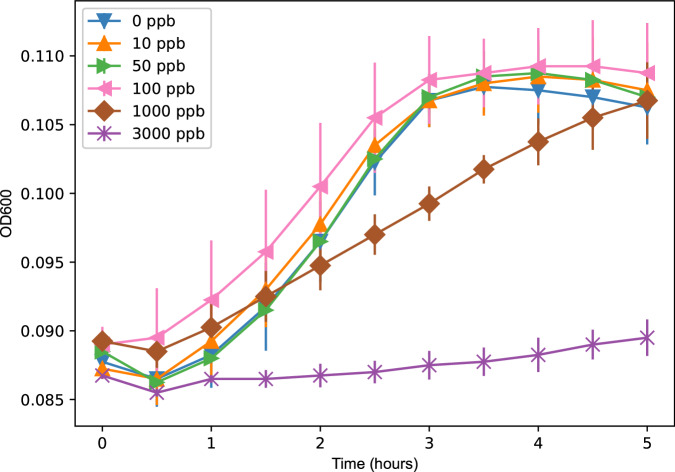
Lead effect on bacterial growth. Lead concentrations varied between 0 and 3000 ppb. Bacteria were growth for 5 h and 37 ^∘^C and monitored at 600 nm. Quadruplicates measurement was carried out in a multiwell plate reader in assay conditions.

In the functional range of our biosensor (0–100 ppb) bacterial growth was not affected. At higher concentrations, notably above 1000 ppb, bacterial growth is affected and a water sample dilution should be made both for bacterial and colour development (Fig. 6). Therefore, we suggest users should dilute their water sample by 1:10, if they suspect that they are evaluating samples with concentrations between 100 ppb and 1000 ppb of lead. Some samples with lead concentrations above 100 ppb were diluted to  < 100 ppb and assayed by the PlomBOX. For instance, a sample with a true lead concentration of 313 ± 63 ppb was diluted to 78 ± 16 ppb. The PlomBOX returned a value of 66 ± 15 ppb, demonstrating its capability to detect initially elevated lead concentrations post-dilution.

Finally, we benchmark the performance of the PlomBOX against AAS, by an initially blind measurement of certified lead solutions. Figure 5 reports the results of these measurements for concentrations ranging from 0 to 100 ppb. Two of these data points, which correspond to 10 and 20 ppb, were re-measured after un-blinding and the final result is the one presented in the figure.

The 95% confidence interval measurements of the PlomBOX are generally in good agreement with the AAS measured values for these real-world water samples. Thus we conclude that, for determination of whether lead concentration exceeds the WHO upper limit, the PlomBOX in the field approximately matches the reliability of AAS in the lab.

## Discussion

This paper reports the design and fabrication of a biological system for environmental monitoring, to help mitigate lead intake through contaminated drinking water by expediting access to assay of lead in water at the point of use. We developed a genetically modified strain of *E. coli* and an imaging system to measure the bacteria response, employing low-cost, commercial off-the-shelf components. We demonstrated the PlomBOX has sensitivity to identify the presence of lead in drinking water in excess of the World Health Organisation upper limit of 10 ppb. We employed the PlomBOX in a household drinking water monitoring campaign in Buenos Aires, and the results demonstrate 83% correlation with the lead concentration of these samples measured via atomic absorption spectroscopy by the National Water Institute in Argentina. These results demonstrate the strong potential of the PlomBOX platform to be an important tool, particularly in developing countries, to reliably test for lead in drinking water at the point of use.

Although greater dilutions could be made for higher concentrations, effective use of the PlomBOX in ranges of more than 10× the WHO limit is not foreseen. To discriminate between the lack of blue colour development due to the death of bacteria compared to low lead concentration, we are developing an internal growth control with another chromophore, to be incorporated and evaluated in future versions of PlomBOX.

**Fig. 7:**
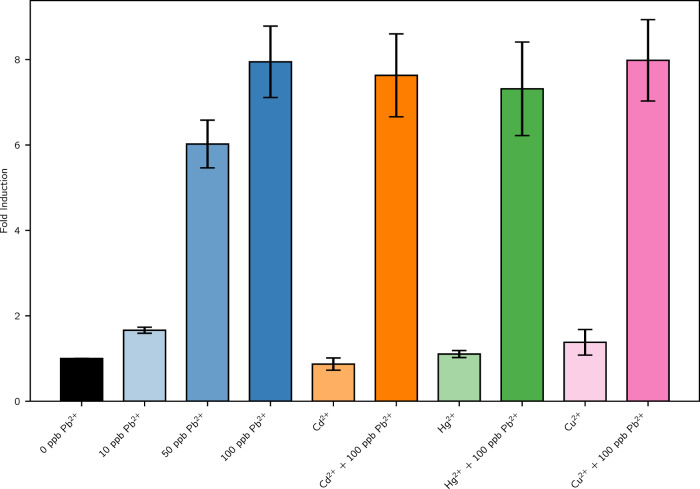
Fold induction for different concentrations of lead and the different interferents, Cadmium (Cd^2+^), Mercury (Hg^2+^) and Copper (Cu^2+^). Each interferent was evaluated at its maximum allowed by the Argentinean Food Code (CAA) with and without lead (0 and 100 ppb of Pb). Four independent assays were conducted, with a duration of 24 h and an incubation temperature of 24 °C. The error bars represent the standard error of the mean of the sample values.

An interference experiment, described in the Specificity measurement section, was conducted to assess if external interference metals affected the bionsensor’s response to lead. Figure 7 shows that this is not the case and Cd^2+^, Hg^2+^ and Cu^2+^ do not interfere with the PlomBOX’s response to lead.

To make the PlomBOX usable by non-experts, we envision disposable cartridges containing lyophilised biosensor bacteria and the X-gal substrate would be distributed, to be loaded and measured in the PlomBOX during a water sampling campaign in the field. This will contribute to environmental management and decision making by regulators. Although lyophilisation in the PlomBOX biosensor is a work in progress, we have already demonstrated the viability of this approach having implemented lyophilised biosensors such as a device for microcystin detection and SensAr (which detects arsenic)^29–31^. Our next goal is to insert the genetic construct into a GRAS microorganism (generally recognised as safe) and integrate it into the genome to avoid the use of antibiotics during overnight incubation of the biosensor for assays.

## Methods

### PlomBOX construction and measurement protocol

#### Bacterial strains, culture conditions and reagents

Laboratory strain *E. coli* DH5*α* was used for transformation with the lead biosensing construct. Transformed bacteria was cultured in LB buffer supplemented with glucose and ampicillin (100 *μ*g mL^−1^) overnight at 37 °C and 220 rpm shaking^32^. X-gal (5-bromo-4-chloro-3-indolyl-*β*-D-galactopyranoside from GenBiotech) was dissolved to 20 mg mL^−1^ in dimethylformamide and stored at  −20 °C. For Luria Bertani/X-gal solution, X-gal was diluted 1/10 in LB medium prepared as in Sambrook 2001^33^. Whatman paper (Whatman Chromatography paper, cat no 3030-6188, grade: 3 mm CHR) was acquired from Cytiva Life Sciences. Lead nitrate 5000 ppb stock was prepared in distilled water using a Lead standard commercial solution of 1000 *μ*g mL^−1^ (Chem-Lab NV) and stored at room temperature. Serial dilutions were made from this stock to prepare the calibration lead solutions.

#### Construction of the lead biosensor

The genetic construction contains the *pbrT* gene (1929 bp, GenBank: CP000354) and the *pbrR* gene (435 bp, Accession Number: AY378100) both under the regulation of the P479 promoter^22^. The divergent promoter region (85 bp, GenBank: AY378100.1) regulates the expression of the LacZ-alpha fragment. This construction was synthesised by GenScript and delivered into the pUC57mini backbone. After transformation with the eluted DNA, plasmid extraction and purification of selected colonies was made. Isolated plasmid DNA was subjected to enzymatic digestions at 37 °C for 1 h and gel electrophoresis to check the release of the correct sized fragments was performed.

#### Construction of the PlomBOX

The electronics system of the PlomBOX comprises a low-cost ESP32-CAM development board and an AD-HOC designed Printed Circuit Board (PCB) which drives 3 colour LEDs^25^.

The ESP32-CAM evaluation board has a CPU, memory chip and the Ominivision OV2640 CMOS colour camera, and supports WiFi and Bluetooth protocols. The camera is used to image the sample to obtain colourimetric information from the biological reporters. Custom firmware was written to control the image sensor and the LED PCB, to communicate via Bluetooth with the mobile phone data acquisition application, interact with the on-board memory and transmit data.

The OV2640 image sensor has a 160 × 1200 pixel array with a Red Green Blue (RGB) colour filter array which provides colour information^34^. The sensor has on-chip sub-sampling which was used to reduce the quantity of transmitted data. It also has on-chip image processing capabilities to improve the perceived visual image quality, including automatic gain control, exposure control, white balance, band filter and black level calibration. These automated features have been disabled to avoid systematic errors in the quantification of the incoming light.

The LED PCB illuminates the sample with a known and reproducible wavelength spectrum of light, thus removing environmental variations from the colour measurement.

#### Mechanical housing

The PlomBOX mechanical housing was designed to be 3D printed; its upper and lower parts come apart to insert the sample for measurement. The enclosure is designed to be light tight, such that the illumination of the sample is dominated by the LED array. The mechanics of the device host the sample holder, comprising 16 wells, at the bottom of the PlomBOX. The top of the PlomBOX supports the electronics and LED board.

The PlomBOX housing and sample holder were printed in a Evo3D EvoI5 FDM printer which uses Polylactic Acid (PLA) plastic (Printalot, PLA 3Di 1.75 mm filament). Liquid leaks were observed between sample holder wells that were printed with PLA. After trying various materials, resin (Hellbot, standard 405 nm, white) was chosen as the optimal material to print the sample holders. The sample holder was printed in resin, using a Hellbot Apolo Pro DLP 3D Printer.

#### Data acquisition application

A mobile phone application, PlomApp, was developed in Android Studio using Java. The application, designed to be functional without additional UI/UX considerations at this stage, controls the PlomBOX through interacting with the firmware running on the ESP32-CAM, acquires image data via Bluetooth and collects meta-data from the smartphone geo-location sensors.

**Fig. 8:**
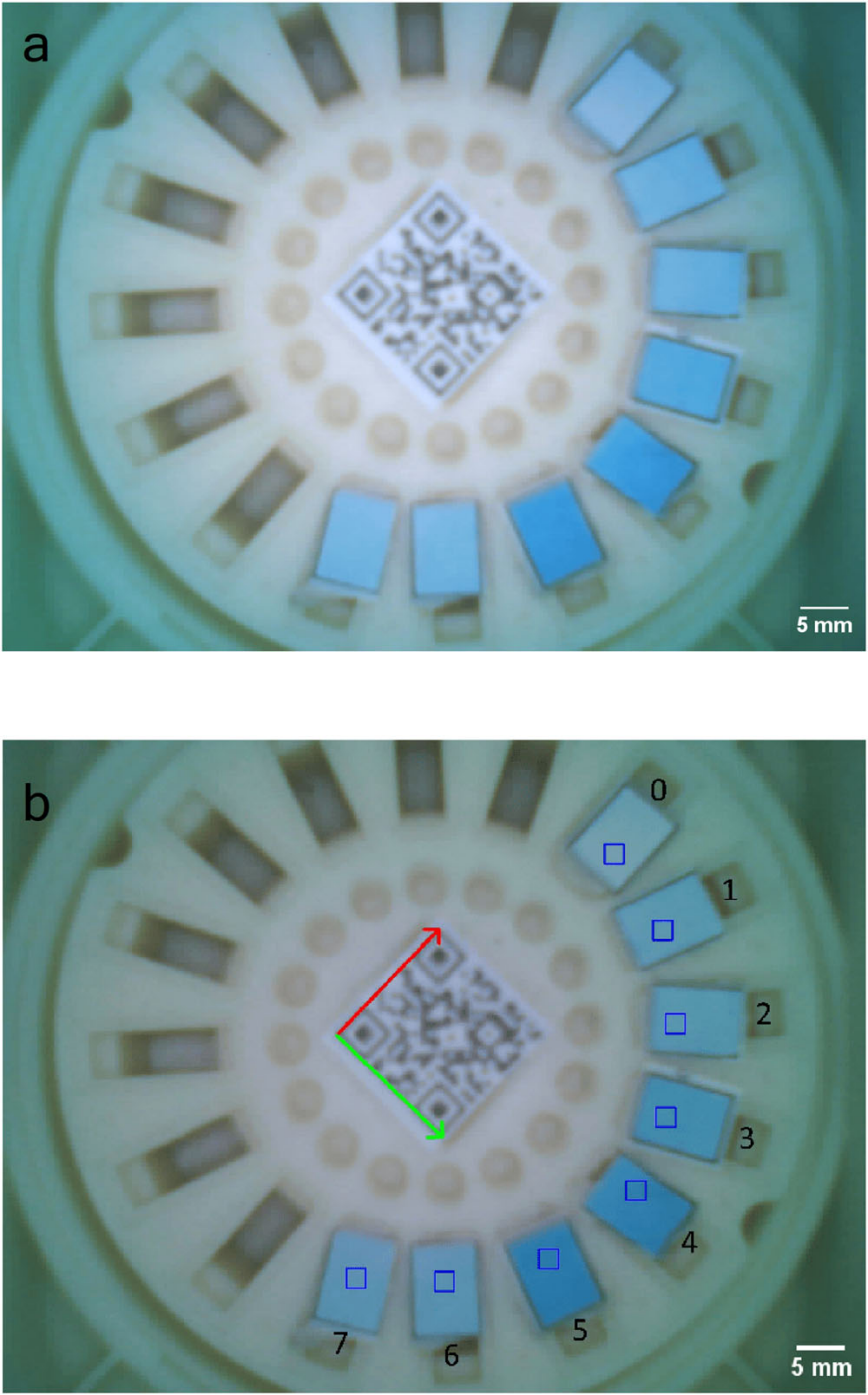
Photo of the PlomBOX’s sample holder, housed inside the PlomBOX. **a**, **b** compare an image of the sample holder before and after flat fielding. ROIs shown on the image as blue squares, labeled from 0 to 7.

Data acquisition begins when the PlomApp sends a command via Bluetooth to the ESP32-CAM to acquire data. A colour calibration image is acquired, followed by a time series of images of the sample holder, as detailed in the Colour measurement and calibration section. Prior to sending data to the phone, images are encoded to byte strings. The data are sent from the ESP32-CAM evaluation board to the PlomApp via Bluetooth; Fig. 8 shows an example image of the sample holder acquired by the PlomApp. Once received on the app, the image data and meta-data are sent to a remote server via MQTT protocol as JSON strings. A secure communication protocol was developed relying on Transport Layer Security certificates for individual users. In the server, data is analysed as described in the Analysis Pipeline section, a lead result per sample is obtained and the result is returned to the PlomApp via MQTT.

#### Measurement duration

The PlomBOX lead assay consists of placing 4 × 14 mm Whatman papers in each well of the sample holder with 12 *μ*L of the PlomBOX bacteria culture incubated overnight, plus 12 *μ*L of LB supplemented with X-gal solution (2 mg mL^−1^ final concentration). Then, 750 *μ*L of the lead calibration solutions (or 750 *μ*L of the water sample to be evaluated) were added to a set of wells which are used to obtain a lead curve. The sample holder is loaded and the PlomBOX device is assembled and connected to the PlomApp.

The sample holder is imaged every 40 min over 17 h (and we also tested imaging every 20 min over 50 h for longer duration studies). Once the timelapse is over, the images are analysed. The blue colour appears first in the wells with the highest concentrations of lead, after approximately 5–6 h at 37 °C (and we also tested longer duration studies at room temperature, where the blue colour appears first after 8–12 h at 24 °C).

To cross-check the PlomBOX measurement fidelity, we compared assay results obtained via the PlomBOX with results obtained from a conventional multiwell plate lead assay strategy. In both cases, overnight incubations of bacteria cultures were done at a temperature of 24 °C and for comparison also at 37 °C. The multiwell plate measurement consists of placing one 10 × 7 mm Whatman paper in each of 24 wells of a multiwell plate, with 12 *μ*L of the overnight PlomBOX bacteria culture in every paper plus 12 *μ*L of LB supplemented with X-gal solution (2 mg mL^−1^ final concentration). Then, 500 *μ*L of the calibration lead solutions or sample water were added to the wells which are used to obtain a calibration curve. The multiwell plate is imaged with a mobile phone CMOS camera.

The multiwell plate measurements enabled qualification of the biosensor bacteria alone, and the comparison of the multiwell image with the PlomBOX timelapse result gave qualitative validation of the biosensor use in the PlomBOX.

#### Specificity measurement

The PlomBOX specificity lead assay was conducted using the same methodology described in the previous section, using 500 *μ*L of the lead curve or the interference solution. Each interfering metal was evaluated at its maximum concentration allowed by the Argentinean Food Code (CAA) with and without Lead (0 and 100 ppb of Pb). The CAA states the maximum concentrations to be 5 *μ*g L^−1^ for Cd^2+^, 1 *μ*g L^−1^ for Hg^2+^ and 1 mg L^−1^ for Cu^2+^. The multiwell plate was incubated overnight at 24 °C. The papers were then positioned on a white base and a picture was taken and analysed, by obtaining the saturation value for each paper. Figure 7 shows the results of 4 independent assays.

### Image data analysis and uncertainty propagation

Following the image colour and uniformity calibrations described in the main body of the paper, which are done via the LED calibration image and flat-fielding steps, the data processing chain applies several calibrations to produce the final measured saturation value for a water sample of unknown lead concentration. These calibrations include an absolute colour calibration in the HSV colour basis, a correction for the lead calibration curve use of de-ionised water rather than tap water and subtraction of the image saturation value at the start of a time series assay measurement. These are described here, together with the related propagation of uncertainties.

#### Image colour and uniformity calibration

Data acquisition begins with an LED calibration run. This entails acquiring three LED calibration images. The 3 RGB LEDs cannot be used at different intensities to construct the calibration image, as it produces banding (due to the hardware pulse-width modulation implementation of the intensity control in the LED ring). We therefore operate them one at a time, at full power, and adjust the exposure time for each of the 3 independent colour images to guarantee a proper white balance. We compute this exposure time by taking a picture of a white paper with a predetermined exposure time (automatic exposure control AEC of 100), and scale the AEC by the ratio between the observed average pixel values and an expected value of 200. The automatic exposure control sets the shutter speed of a camera based on the external lighting conditions^35^.

The average colour in the centre of each white paper RGB image (${\overline{RGB}}_{{{\rm{meas}}}}$) is used to obtain the calibration values of the LEDs, via

1 $$C(R,G,B)=AEC\times \frac{\overline{RGB}}{{\overline{RGB}}_{{{\rm{meas}}}}}=100\times \frac{200}{{\overline{RGB}}_{{{\rm{meas}}}}}$$

An AEC of 100 was assumed for the assays discussed in this paper and corresponds to an expected average value given the illumination of the hardware we use but, in principle, this value can be adjusted should the PlomBOX be made of a different material.

Following this step, the white paper is removed and data is acquired for the sample holder. For each data set, three images, illuminated sequentially by R, G, or B LEDs, are acquired.

When data acquisition is completed, these calibrated images are sent to the server to be analysed.

On the server, each set of R, G, B calibrated images are merged to obtain a single LED calibration image and a single LED calibrated image. This allows for the calibrated image to be subsequently flat fielded, as

2 $${I}_{{{\rm{C}}}}=\frac{{I}_{{{\rm{R}}}}\cdot {i}_{m}}{{I}_{{{\rm{F}}}}}$$

where *I*_C_ is the corrected image, *I*_R_ is the raw (LED-calibrated) image, *I*_F_ is the flat field (LED calibration) image and *i*_*m*_ = $\overline{RGB}$ = 200 corresponding to the colour of a piece of white paper. The resultant flat fielded image can be seen in Fig. 8, showing how the heterogeneous illumination of the sample holder has been removed.

After flat fielding, the analysis script uses the QR code seen on the image to find the ROIs where the bacteria samples are placed, and HSV values are calculated from the average RGB values of the image as described in the Analysis Pipeline section. For the example shown in Fig. 8b, the RGB, HSV and their uncertainty values, *σ*_R_, *σ*_G_, *σ*_B_, *σ*_H_, *σ*_S_ and *σ*_V_, are shown in Table 4.

**Table 4:** RGB (Red, Green, Blue), HSV (Hue, Saturation, Value) and their uncertainties for each ROI shown in Fig. 8

ROI	R	G	B	*σ*_R_	*σ*_G_	*σ*_B_	H	S	V	*σ*_H_	*σ*_S_	*σ*_V_
0	116.3	159	172.8	0.2	0.2	0.5	194.7	32.7	67.8	0.3	0.3	0.2
1	90.6	151.1	173.9	0.2	0.2	0.2	196.4	47.9	68.2	0.2	0.2	0.1
2	87.8	149.6	174.7	0.1	0.1	0.4	197.3	49.7	68.5	0.2	0.2	0.1
3	66.2	140.2	173	0.2	0.2	0.2	198.4	61.7	67.8	0.1	0.1	0.1
4	45.6	126.9	170	0.3	0.4	0.6	200.8	73.2	66.7	0.2	0.4	0.2
5	40.8	122.3	172.4	0.1	0.1	0.4	202.9	76.3	67.6	0.1	0.3	0.2
6	90.6	143.9	175.7	0.4	0.2	0.3	202.4	48.4	68.9	0.3	0.3	0.1
7	92.8	144.3	176.3	0.3	0.2	0.5	203	47.4	69.2	0.2	0.4	0.2

The RGB uncertainties are obtained by calculating the standard error of the mean of R, G and B values, ${\sigma }_{\overline{j}}$, for all the pixels in each ROI:

3 $${\sigma }_{\overline{j}}=\frac{{\sigma }_{j}}{\sqrt{{n}_{p}}}\,\,{\mbox{for}}\,\,j\,\in \,\,{\mbox{R},\,\mbox{G},\,\mbox{B}}$$

where *σ*_*j*_ is the sample standard deviation for R, G and B and *n*_*p*_ is the number of pixels. The chroma, Ch, and HSV uncertainties are obtained from standard error propagation.

#### Hue, saturation and value calibration

The colour measurement analysis was applied to samples of known colour composition to validate the analysis method, and obtain absolute colour calibration constants such that operators of PlomBOX devices in different locations, with different lighting conditions, can meaningfully compare with each other.

**Fig. 9:**
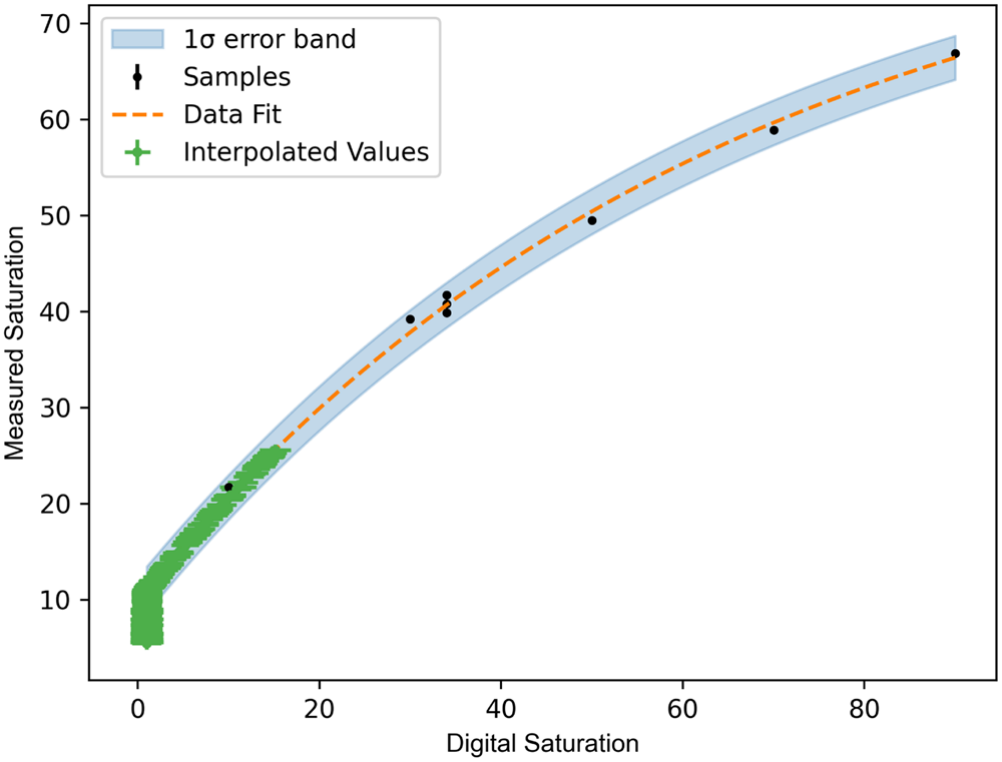
Digital saturation vs measured saturation with an exponential fit for sample ROIs. The measured saturation values for samples, in green, are interpolated to determine their digital saturation values.

A wheel of ROIs was coloured digitally with known RGB (and HSV) values. This was printed onto a paper, placed inside the PlomBOX and images were acquired. The saturation values for each ROI were measured following the analysis described in the main body of the paper, and compared with the digital saturation values used to colour the ROIs. Figure 9 shows the digital saturation values compared with the measured saturation values. The data in Fig. 9 are fit with the function *y* = *a* ⋅ *e*^−*b*⋅*x*^ + *c*, where *y* is the measured saturation, *x* is the digital saturation and *a*, *b* and *c* are the three free parameters in the fit. This calibration curve is used in subsequent data analysis: measured saturation values are interpolated using this function to determine their digital saturation values. This is done such that the saturation value at the end of an assay has a meaning that can be compared between PlomBOX devices operated at different sites, under different illumination conditions.

The uncertainties coming from this calibration are included in the uncertainty propagation as follows. The fit function was rearranged to find x,

4 $$x=-\frac{\ln \left(\frac{y-c}{a}\right)}{b}$$

and the error propagated via standard error propagation.

This HSV calibration relative to digital colour values can be done for each PlomBOX unit used, to remove variations in imaging conditions etc.

#### Time series first image subtraction

As described in the main body of the paper, a time series of images is obtained to measure the growth of the bacteria in response to lead. To remove any variations between assays occurring at different times, the first image is subtracted from all subsequent images in the time series, such that the starting value of saturation in each assay is zero.

The uncertainties on the subtracted saturation values are propagated as:

5 $${\sigma }_{{{\rm{sub}}}}=\sqrt{{\sigma }_{\#1}^{2}+{\sigma }_{\#N}^{2}}$$

Where *σ*_sub_ is the uncertainty of the subtracted saturation, *σ*_*#*1_ is the uncertainty of the saturation of the first image on the timelapse and *σ*_*#*N_ is the uncertainty of the saturation of the image being subtracted.

#### Time series data fit function and uncertainty propagation

As described in the main body of the paper, the lead measurement for a sample of unknown concentration is based on fitting the saturation vs. time obtained from a time series of images of the sample holder within the PlomBOX.

The fit function chosen includes two sigmoid functions (or Monod curves): one to represent the gene expression and one to represent the continuous bacterial growth throughout the assay. The Monod equation models the growth of bacteria populations^36^. It is dependent on the growth rate of the bacteria, *μ*, and on the limiting substrate for growth, *S*, which in this case is the LB broth for the curve which represents the bacterial growth and is X-Gal for the curve which represents the gene expression:

6 $$\mu ={\mu }_{\max }\frac{S}{{K}_{{{\rm{S}}}}+S}$$

Here, ${\mu }_{\max }$ is the maximum growth rate for the bacteria and *K*_S_ is the value of the substrate when $\frac{\mu }{{\mu }_{\max }}=0.5$. The Monod curve has five distinct phases: the lag phase at the beginning, which occurs until the bacteria have acclimated to their new environment; the exponential growth phase; the deceleration phase, when the limiting substrate starts to become depleted; the stationary phase, when the net bacteria growth is approximately zero and the death phase when the bacteria are destroyed by lysis, i.e. when the bacteria walls are broken down or destroyed^37^. The first four phases are observed on data acquired for all assays.

The fit function is

7 $$y=\frac{a}{1+{e}^{(-t+b)\cdot (\frac{c}{1+{e}^{-t}})}}$$

where $\frac{a}{1+{e}^{(-t+b)}}$ relates to the gene expression, and $(\frac{c}{1+{e}^{-t}})$ relates to the bacterial growth. The *a* parameter represents the amplitude of the curve, i.e. the maximum saturation that can be obtained, *b* is the value of *t* when *y* has reached 50% of its final value and *c* represents the slope of the curve. *t* is time, in minutes, and *y* is saturation.

The uncertainty on the final saturation value, which is the best-fit function value evaluated at the end time of the assay, is calculated accounting for the uncertainties on the fit parameters *σ*_a_, *σ*_b_ and *σ*_c_ as

8 $${\sigma }_{{{\rm{sat}}}.}=\sqrt{\sum _{j=a}^{b,c}\left\{{\left(\frac{\partial y}{\partial j}\right)}^{2}\cdot {\sigma }_{j}^{2}\right\}\quad +\sum _{k\,l=a\,b}^{a\,c,b\,c}\left\{\left(\frac{\partial y}{\partial k}\right)\left(\frac{\partial y}{\partial l}\right)\cdot {\sigma }_{k}{\sigma }_{l}\right\}}$$

where

9 $$\begin{array}{rcl}\frac{\partial y}{\partial a}&=&\frac{1}{1+{e}^{\frac{c\cdot (b-t)}{1+{e}^{-t}}}}\\ \frac{\partial y}{\partial b}&=&\frac{-a\cdot c\cdot {e}^{\frac{c\cdot (b-t)}{1+{e}^{-t}}}}{{\left(1+{e}^{\frac{c\cdot (b-t)}{1+{e}^{-t}}}\right)}^{2}\cdot (1+{e}^{-t})}\\ \frac{\partial y}{\partial c}&=&-\frac{a\cdot {e}^{\frac{c\cdot (b-t)}{1+{e}^{-t}}}\cdot (b-t)}{{\left(1+{e}^{\frac{c\cdot (b-t)}{1+{e}^{-t}}}\right)}^{2}\cdot (1+{e}^{-t})}\end{array}$$

The goodness of the fit is quantified with a reduced chi-square statistic.

Figure 3 provides an example of a timelapse fit, showing the sigmoid fits, the 1 *σ* error band and the ${\chi }_{{{\rm{red}}}}^{2}$ value for this fit.

#### Lead curve fits

Samples of known lead concentration are co-measured with the unknown samples to calibrate the bacteria response. To interpolate between measured saturation values to form a lead curve, a first-order polynomial *y* = *ax* + *b* is fit to the saturation vs. lead concentration data for the known samples. Here *x* is lead concentration, in ppb, *y* is saturation, and *a* and *b* are the fit parameters, the slope and the saturation-axis intercept respectively.

The saturation values of the unknown tap water samples are linearly interpolated on this fit, returning a value of lead for each of them. The uncertainty contribution from the lead curve calibration fit is propagated as follows. The lead value *x* is

10 $$x=\frac{y-b}{a}$$

and its uncertainty is obtained with a similar method as described in Equation (8).

Figure 4 shows an example lead curve fit, with lead curve data points, interpolated sample data, the best-fit function, and the 1 *σ* error band calculated as described here.

#### Data quality cuts

Data quality cuts are applied following the analysis of all assays to select assays in which the bacteria successfully grew.

The *b* parameter of the timelapse fit, Equation (7), which is the time when the saturation has reached 50% of its final value, was checked for all timelapse curves and a cut was defined in which an assay’s data was excluded if the *b* parameter  >1000 min. Observing $b \, > \, 1000\,\min$ indicates that the biosensor did not grow successfully for that assay.

This cut was applied to all samples of known concentrations in the lead curve, as well as to samples of unknown concentration. If more than one element of a lead curve did not pass the cut, the entire assay was disqualified. Additionally, any individual samples which did not pass the cuts were excluded from the analysis described in the PlomBOX Accuracy section.

A further cut was applied to address instances in which the saturation of the 100 ppb element of the lead curve was lower than the saturation corresponding to 50 ppb. The growth of *E. coli* can be inhibited when in the presence of media with large lead concentrations. When the saturation for 100 ppb is lower than the saturation for 50 ppb, the 100 ppb point is excluded from the lead curve fits.

Two of six assays, each of which hosted 6 calibration samples and 9 water samples, passed the selection criteria for the water sampling campaign. Seven of thirteen assays passed the criteria for the milli-Q campaign.

### Analysis results

#### PlomBOX accuracy

As described in the main body of the paper, accuracy is assessed by measuring samples of known concentration multiple times and calculating the 95% confidence interval. Table 1 (Table 3) shows the CI and *p*-values obtained for various lead concentrations using milli-Q water (de-ionised water), reported graphically in Fig. 5.

### PlomBOX validation against external measurements

#### Tap vs. De-ionised Water

The validity of PlomBOX measurements with tap water was checked, as our measurements under real-world conditions were conducted with tap water, whereas our laboratory measurements are conducted with de-ionised water. We deem this necessary because the bacteria growth in tap water can be impeded by the trace presence of disinfectants, i.e. chlorine^38^. A lead curve was created adding lead to tap water and it was compared with a corresponding curve obtained with de-ionised water. This was to the end of establishing a calibration constant, allowing to convert between the two curves.

The full data processing analysis was applied to both, and measured saturation values were obtained for lead concentrations of 0, 10, 50, 100, 200 and 500 ppb. Both curves were fitted with an exponential fit; these fits were compared and a ratio,  ≈ 1.08, was obtained as a function of lead concentration. The de-ionised lead curve saturation values are divided by this ratio to obtain saturation values representative of a tap water lead curve.

The uncertainties on the tap water saturation values are propagated, as

11 $${\sigma }_{{{\rm{tap}}}}={S}_{{{\rm{tap}}}}\cdot \frac{{\sigma }_{{{{\rm{dH}}}}_{2}{{\rm{O}}}}}{{S}_{{{{\rm{dH}}}}_{2}{{\rm{O}}}}}$$

where *S*_tap_ is the tap water saturation value, ${\sigma }_{{{{\rm{dH}}}}_{2}{{\rm{O}}}}$ is the uncertainty and ${S}_{{{{\rm{dH}}}}_{2}{{\rm{O}}}}$ the value of saturation for de-ionised water.

A user in a different laboratory should repeat this exercise for the water they assay, as it will potentially contain different constituents. However we note that this correction is at the  < 10% level.

#### INA sampling campaign and chemical analysis

Drinking water samples were taken from selected houses in Buenos Aires suspected of containing lead plumbing, as follows. A total of 66 samples were taken for domiciliary water monitoring during three campaigns. One sample was taken outside the house connected directly to the main water company distribution pipes. This sample should represent a control with good water quality. Then, inside the house, three more samples were taken from the kitchen. Before the sample collection, residents were requested not to open any tap for 24 h to let the still water accumulate lead from the pipes. The first sample is taken immediately after opening the tap collecting the sample into a plastic container. The second sample of water is collected one minute after the tap was left running to ensure a complete purge of the plumbing connection to the water tank. A third sample is taken after leaving the water running for a further five minutes. Once obtained, the samples are divided: one for analysis with traditional analytical methods, *i.e*. AAS, and preserved with nitric acid, while the other is measured by the PlomBOX.

Lead analysis were performed at INA using the standard analytical protocol following Spectophotometry of Atomic Absorption (Shimadzu, Japan) with a graphite furnace according to standard methods (SM 3113 A and B; APHA, 2017). Drinking water samples were analysed directly without a pre-digestion step. A 1000 mgPb L^−1^ standard stock solution (Pb/(NO3)2; Spectro ECON ChemiLab p.a.) was prepared with 5% Nitric acid, and then diluted to prepare the analytical solutions ranging from 0.005–0.030 mgPb L^−^^1^, corresponding to 5–30 ppb. Detection and quantification limits of this process are 0.02 and 0.05 mgPb L^−1^, respectively. High purity Millipore water (Millipore*Ⓡ*, Merck, Germany) was used throughout the process. All the glass receptacles for the Pb analysis and bottles used for water sampling, were thoroughly washed with 50% nitric acid solution to effectively remove any metal traces.

The correlation between the PlomBOX measured lead values and the AAS results for the water samples collected in the assay campaign was quantified using the Pearson correlation coefficient. In these measurements, the lead curve samples are produced using de-ionised water. This is done because de-ionised water is more readily available in the field than milli-Q ultrapure water, and using de-ionised water eliminates potential variations between tap water available in different laboratory sites.

## Data Availability

The data needed to produce the PlomBOX measurement platform have been deposited in the open source GitHub repository https://gitlab.com/plombox/open-science.

## Materials availability

The materials needed to produce the PlomBOX measurement platform have been deposited in the open source GitHub repository https://gitlab.com/plombox/open-science.

## Code availability

The code needed to produce the PlomBOX measurement platform has been deposited in the open source GitHub repository https://gitlab.com/plombox/open-science.
